# Inpatient Expenditure Composition and Factors Associated with High-Expenditure Patients with Chronic Wounds: A Retrospective Analysis

**DOI:** 10.3390/healthcare14193252

**Published:** 2026-10-01

**Authors:** Yishan Zhou, Aijun Lu, Xuemei Wang, Yufeng Jiang

**Affiliations:** 1 Public Health College, Inner Mongolia Medical University, Hohhot 010010, China; 15548351619@163.com (Y.Z.); wangxm_zsu@163.com (X.W.); 2 Affiliated People’s Hospital of Inner Mongolia Medical University, Hohhot 010017, China; luaijun2026@163.com; 3 Department of Tissue Regeneration and Wound Repair, The First Medical Center, Chinese PLA General Hospital, Beijing 100853, China

**Keywords:** chronic wounds, inpatient expenditure, expenditure drivers, high-expenditure patients, healthcare economics, retrospective study

## Abstract

**Background**: Chronic wounds impose a substantial clinical and economic burden on patients and health systems. The purpose of this study was to characterize the composition and concentration of inpatient expenditure among patients with chronic wounds, identify clinical and treatment-related factors associated with higher inpatient expenditure, and describe the characteristics and expenditure contribution of high-expenditure patients. **Methods**: This single-center retrospective study included 713 patients discharged after inpatient treatment for chronic wounds at a tertiary hospital in the Inner Mongolia Autonomous Region, China, between 2020 and 2024. Demographic characteristics, comorbidities, infection status, wound pathogen results, treatment procedures, clinical outcomes, length of stay, and inpatient expenditure were collected. Patients in the top 10% of total inpatient expenditure were defined as high-expenditure patients. Descriptive analyses, expenditure stratification, multivariable regression, and expenditure concentration analyses were performed. **Results**: Medical consumables, diagnostic services, treatment procedures, and medications were the main components of inpatient expenditure. Length of stay, peripheral arterial disease, clinical infection, cardiovascular disease, comorbidity burden, wound pathogen results, and surgical interventions were associated with higher inpatient expenditure. High-expenditure patients represented a small proportion of the cohort but accounted for approximately one-third of total inpatient expenditure and had greater clinical complexity, including higher prevalence of infection and vascular disease and more intensive treatment needs. **Conclusions**: Inpatient chronic wound care is characterized by substantial expenditure, complex clinical management, and concentration of resource use among a small high-expenditure subgroup. Early risk identification, multidisciplinary management, standardized treatment pathways, and targeted resource-allocation strategies may help improve the efficiency of chronic wound care.

## 1. Introduction

Chronic wounds are wounds that fail to progress through the normal healing process in a timely manner because of local or systemic factors. They have become an important global public health concern and are closely associated with population aging, diabetes, vascular disease, infection, prolonged pressure, and multimorbidity [1,2,3]. A systematic review reported a pooled prevalence of mixed-etiology chronic wounds of approximately 2.21 per 1000 population, indicating a non-negligible epidemiological burden [1]. Chronic wounds often require long-term dressing changes, repeated debridement, antimicrobial treatment, vascular assessment, and revascularization. In severe cases, patients may require amputation or combined procedures. Their management is therefore complex and commonly involves multidisciplinary collaboration among wound-care specialists, endocrinologists, vascular surgeons, infectious disease physicians, nutrition teams, and rehabilitation professionals [3,4,5]. Compared with acute wounds, chronic wounds are characterized by prolonged disease courses, high recurrence rates, long treatment duration, low healing rates, and sustained health-resource utilization.

Inpatient treatment often accounts for a major component of direct medical expenditure [4,5,6]. Previous economic studies have shown that chronic wounds generate substantial health-system costs, although the magnitude and composition of these costs vary across countries, care settings, and payment systems. In the United States, Nussbaum et al. evaluated the aggregate cost and Medicare policy implications of chronic non-healing wounds among Medicare beneficiaries, highlighting the considerable economic importance of chronic wound care. In the United Kingdom, Guest et al. estimated that the National Health Service managed approximately 3.8 million patients with wounds in 2017/2018, with an annual wound-management cost of approximately GBP 8.3 billion; inpatient expenditure was markedly higher for unhealed wounds than for healed wounds [6]. Diabetic foot ulceration is an important subtype of chronic wounds and carries a substantial economic burden. In the United States, the total estimated cost of diagnosed diabetes was USD 412.9 billion in 2022, including USD 306.6 billion in direct medical costs and USD 106.3 billion in reduced productivity [7]. Another study reported that the direct cost of diabetes and its complications in the United States in 2007 was at least USD 116 billion, of which at least 33% was related to the treatment of foot ulcers [8]. In England, the cost of diabetic foot ulcers and amputations in 2014–2015 was estimated at GBP 837–962 million, accounting for 0.8–0.9% of the NHS budget [9]. Rice et al. also reported that diabetic foot ulcers were associated with increased healthcare utilization and additional annual costs among both Medicare and privately insured populations [10]. Together, these studies indicate that the economic burden of chronic wounds is not limited to wound dressings or medications, but also includes outpatient visits, inpatient care, procedures, nursing care, long-term management, and complications such as infection and amputation [3,4,5]. Previous studies have identified clinical complexity as an important contributor to higher wound-care expenditure. In the EURODIALE study, Prompers et al. [11] examined resource utilization and costs among patients with diabetic foot ulcers and showed that peripheral arterial disease and infection were important indicators of greater clinical complexity and resource use. Severe infection, peripheral arterial disease, diabetes, renal insufficiency, cardiovascular disease, and repeated surgical interventions may prolong hospitalization and be associated with higher treatment expenditure. These findings suggest that the economic burden of chronic wound care is closely linked to disease severity, comorbidity burden, impaired perfusion, infection control, and treatment intensity.

In addition to individual clinical factors, previous studies have shown that healthcare expenditure is often concentrated among a small subgroup of high-cost users. Wodchis et al. found that high-cost users accounted for a small proportion of the population but a disproportionately large share of health expenditures, with chronic disease, infection, acute events, and palliative care being common reasons for hospitalization among these users [12]. Holle et al. reported that the top 4.6% of spenders in the United States accounted for 50% of healthcare expenditures in 2018, demonstrating that expenditure concentration is common in high-income health systems [13]. Similar patterns have been observed in inpatient diabetic foot ulcer care, where the top 10% of patients contributed disproportionately to total inpatient expenditure and had greater comorbidity burden, more complex treatment, and poorer outcomes [14]. However, whether this pattern of expenditure concentration also applies to broader inpatient chronic wound populations remains insufficiently explored. Patients with chronic wounds may be particularly vulnerable to expenditure concentration because their care commonly involves prolonged hospitalization, repeated dressing changes, microbiological testing, antimicrobial therapy, vascular assessment, surgical debridement, revascularization, amputation, and multidisciplinary management [15,16,17]. These clinical features may lead to repeated procedures, intensive use of medical consumables, longer length of stay, and complex treatment pathways. Therefore, although expenditure concentration has been well established in general healthcare populations, further investigation is needed in the specific context of inpatient chronic wound care.

The Chinese healthcare system differs from many Western settings in terms of hospital-centered service delivery, insurance reimbursement arrangements, regional variation in medical resources [17,18], and ongoing payment reforms, including diagnosis-related group and diagnosis-intervention packet payment reforms. In China, patients with chronic wounds are often managed in tertiary hospitals when they have infection, ischemia, severe comorbidities, or complex surgical needs. Hospital-level inpatient expenditure data can therefore provide important information for identifying patients at risk of high expenditure, optimizing inpatient treatment pathways, improving resource allocation, and supporting expenditure-control strategies under the Chinese healthcare system. However, evidence on inpatient expenditure for chronic wound care in China remains limited. Existing studies have focused mainly on diabetic foot ulcers, national cost estimates, overall medical costs, or general high-cost user populations [19]. Relatively few studies have examined the detailed composition of inpatient expenditure among patients with chronic wounds, the clinical and treatment-related factors associated with higher expenditure [20], or the concentration of expenditure among high-expenditure patients in Chinese hospital settings. In the context of DRG/DIP-related payment reforms and increasing emphasis on hospital cost control [21], further hospital-based evidence is needed to clarify how inpatient expenditure is distributed, which factors are associated with higher expenditure, and whether a small subgroup of patients accounts for a disproportionate share of total inpatient expenditure.

Accordingly, the purpose of this study was to characterize inpatient expenditure among patients hospitalized for chronic wounds at a tertiary hospital in the Inner Mongolia Autonomous Region of China. Specifically, the objectives were to: (1) describe the composition and concentration of inpatient expenditure; (2) identify clinical and treatment-related factors associated with higher inpatient expenditure; and (3) characterize high-expenditure patients and quantify their contribution to total inpatient expenditure.

## 2. Materials and Methods

### 2.1. Study Design and Participants

This was a single-center retrospective observational study conducted at a tertiary hospital in the Inner Mongolia Autonomous Region, China. The study was reported in accordance with the Strengthening the Reporting of Observational Studies in Epidemiology (STROBE) statement [22]. The study population comprised patients who were hospitalized and discharged after chronic wound treatment between 1 January 2020 and 31 December 2024.

For the operational purpose of this retrospective inpatient study, potentially eligible inpatient records were initially identified using chronic wound-related ICD-10 diagnostic codes. Because ICD-10 codes alone may not accurately capture all chronic wound conditions, final eligibility was determined through medical-record verification, including review of wound-related diagnoses, clinical documentation, treatment procedures, and inpatient expenditure records. Included wound types comprised diabetic foot ulcers, pressure injuries, venous ulcers, arterial ulcers, iatrogenic or postoperative non-healing wounds, traumatic chronic wounds, infection-related chronic wounds, and other chronic wounds [3,4,23].

**Figure 1:**
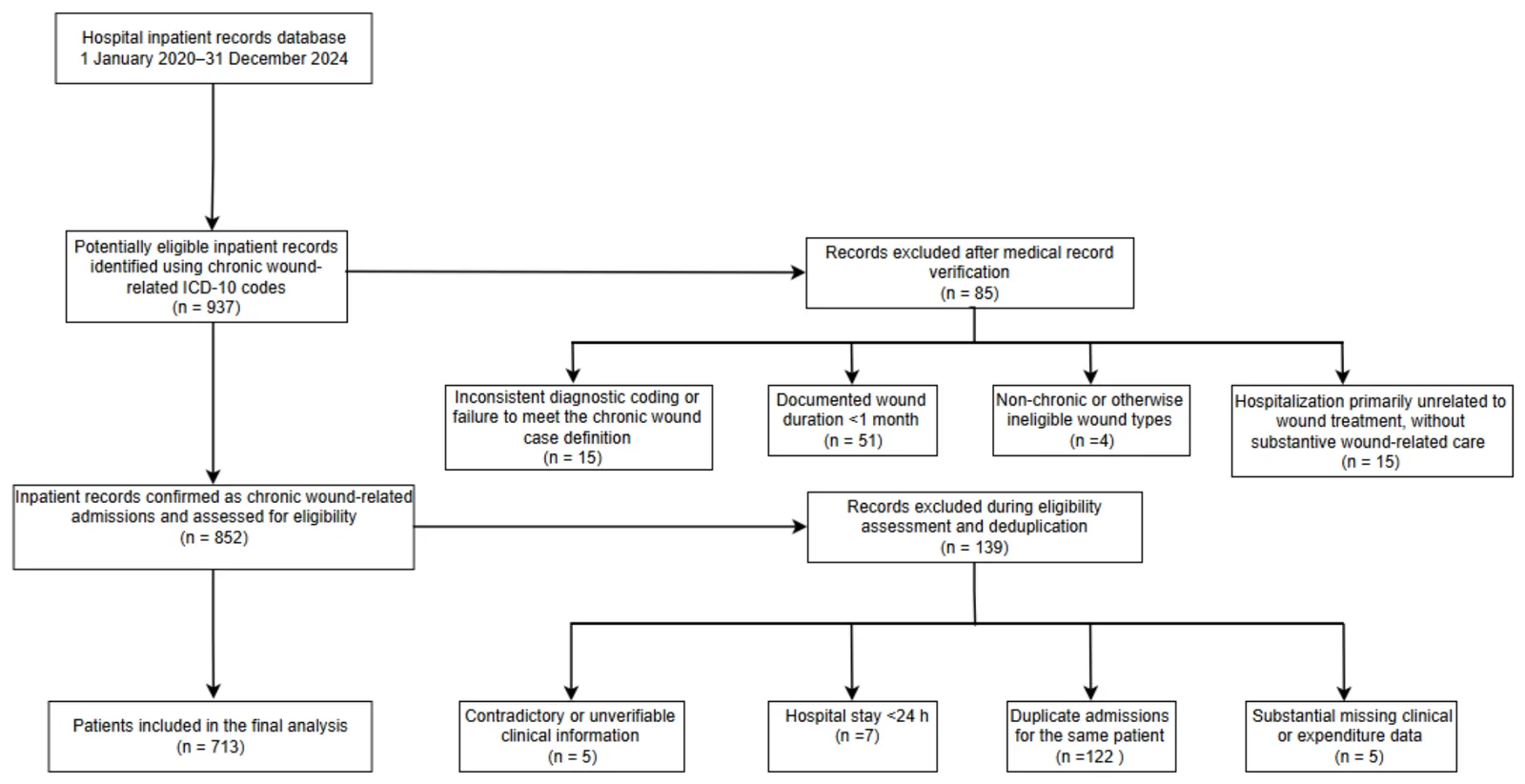
Flow diagram of case identification, eligibility assessment, exclusions, and final inclusion in the analytic cohort. ICD-10, International Classification of Diseases, 10th Revision.

Patients were identified using the International Classification of Diseases, 10th Revision (ICD-10) diagnostic coding system used in the hospital medical-record system. The case identification and selection process is summarized in Figure 1. Potentially eligible inpatient records were initially identified using chronic wound-related ICD-10 codes. These records were further reviewed through medical record verification, including admission information, present illness history, progress notes, discharge diagnoses, wound-related procedures, and inpatient expenditure records. Records were excluded according to predefined eligibility criteria, and duplicate admissions from the same patient were removed before establishing the final analytic cohort. The specific diagnostic names and ICD-10 codes used for case identification are reported in Appendix A, Table A1. To improve coding validity, the initially extracted cases were further reviewed by cross-checking ICD-10 discharge diagnosis codes with electronic medical records, wound-related clinical diagnoses, treatment procedures, and inpatient expenditure records. Cases with inconsistent diagnostic coding, substantial missing information, or no substantive wound-related diagnostic or therapeutic procedures were excluded from the final analytic cohort. Additional exclusions identified during medical record verification included contradictory or unverifiable clinical information, duplicate admissions, and non-chronic or otherwise ineligible wound types. ICD-10 codes were used as a screening algorithm rather than a definitive diagnostic criterion. Previous studies have demonstrated that administrative coding algorithms for diabetic foot ulcers and related wound conditions require validation against clinical records because coding accuracy varies according to the algorithm used and the data source [24]. Therefore, in the present study, ICD-10 retrieval was followed by manual clinical verification to improve case ascertainment accuracy.

### 2.2. Inclusion and Exclusion Criteria

The inclusion criteria were as follows: (1) an anatomically identifiable wound was explicitly documented in the medical record; (2) meeting the evidence-based clinical definition of chronic wounds according to international wound-care literature and consensus statements [3,4,23], together with medical-record documentation of persistent or delayed wound healing for more than one month; (3) chronic wound assessment or treatment constituted a substantive component of the hospitalization; and (4) the wound diagnosis and anatomical site were supported by admission notes, progress notes, discharge diagnoses, wound-care documentation, operative records, or other relevant clinical evidence. Isolated codes for infection, gangrene, peripheral arterial disease, trauma, burns, frostbite, or postoperative complications were insufficient for case confirmation in the absence of documented evidence of a persistent wound. Diabetic foot ulcer was not defined by a single ICD-10 code. A case was classified as a diabetic foot ulcer only when the medical record documented both: (1) diabetes mellitus and (2) an ulcer involving the foot. Diabetes-related codes indicating peripheral neuropathy, peripheral arterial disease, infection, gangrene, or other foot disease without explicit documentation of a foot ulcer were not considered sufficient for diabetic foot ulcer classification. The classification approach was informed by previously validated administrative-data algorithms and the IWGDF definitions for diabetes-related foot disease [24]. The exclusion criteria were as follows: (1) hospital stay shorter than 24 h; (2) wound duration shorter than one month; (3) substantial missing data; and (4) hospitalization primarily for reasons unrelated to wound treatment. The fourth criterion was determined using a standardized review procedure based on the principal discharge diagnosis, the hierarchy of ICD-10 discharge diagnosis codes, wound-related secondary diagnoses, wound-related diagnostic or therapeutic procedures, and inpatient expenditure records. Patients were excluded only when the principal reason for admission was clearly unrelated to wound care and when the hospitalization did not include substantive wound-related diagnostic or therapeutic procedures. Patients with multimorbidity were not excluded if chronic wound assessment or treatment constituted a major component of the admission. The identification, medical-record verification, deduplication, eligibility assessment, and final inclusion of patients are summarized in Figure 1, consistent with recommendations for transparent reporting of participant selection in observational studies [24].

### 2.3. Variables Collected

The following variables were collected: (1) demographic data, including age and sex; (2) clinical data, including primary diagnosis, comorbidities, modified Charlson Comorbidity Index (mCCI), clinical infection status, and wound pathogen results; (3) treatment data, including debridement/drainage, vascular angioplasty, minor amputation, major amputation, combined surgery, and the number of chronic-wound-related procedures; (4) outcome indicators, including length of stay, clinical outcome, and 30-day chronic-wound-related readmission; and (5) expenditure data, including total inpatient expenditure, comprehensive medical service fees, diagnostic fees, treatment fees, medication fees, blood and blood-product fees, medical consumable fees, and other fees.

Comorbid conditions were identified using the ICD-10 coding algorithms developed by Quan et al. (2005) and assigned the updated weights reported by Quan et al. (2011) [25,26,27]. A modified Charlson Comorbidity Index (mCCI) was constructed to quantify overall comorbidity burden while minimizing overlap with comorbid conditions entered separately as predictor variables. Charlson components corresponding to diabetes mellitus, peripheral vascular disease, renal disease, and cardiovascular conditions overlapping with the separately defined cardiovascular disease variable were excluded from the mCCI. Age points were also excluded because age was entered separately as a continuous covariate. All remaining Charlson components retained the updated weights reported by Quan et al. (2011) [28], with the original hierarchy rules applied where appropriate. The resulting mCCI was analyzed as a continuous variable and was entered in the multivariable models together with age, diabetes mellitus, peripheral arterial disease, renal insufficiency, and cardiovascular disease.

Inpatient expenditure data were extracted from the hospital information system and were based on itemized inpatient billing records. These data represented charges recorded during hospitalization, including medical consumables, diagnostic services, treatment procedures, medications, nursing care, bed charges, and other inpatient billing items. Therefore, the expenditure data in this study reflect hospital billing expenditure from the inpatient care episode rather than the true economic cost of care, insurer reimbursement, patient out-of-pocket payments, or societal costs. The analysis was conducted from a hospital inpatient billing perspective. Data on insurance reimbursement, patient self-payment, outpatient care, post-discharge care, rehabilitation, productivity loss, and family caregiving costs were not available in the retrospective dataset.

### 2.4. Definitions of Clinical Infection Status and Wound Pathogen Results

Clinical infection status and wound pathogen results were recorded as separate variables. Clinical infection was defined as a documented wound-related infection diagnosis supported by clinical evidence of local or systemic inflammatory manifestations. Local signs included erythema, warmth, swelling, pain or tenderness, purulent discharge, malodor, delayed wound healing, wound deterioration, or surrounding cellulitis. Systemic or laboratory evidence included fever, elevated white blood cell count, elevated C-reactive protein, or other inflammatory markers when available. This definition was aligned with international wound-infection principles, including the International Wound Infection Institute consensus and the IWGDF/IDSA diagnostic principles for diabetes-related foot infection, which emphasize that wound infection is primarily a clinical diagnosis rather than a diagnosis based solely on culture positivity [29,30].

Because this was a retrospective study, infection status was extracted from existing electronic medical records, discharge diagnoses, wound-related clinical notes, laboratory findings, and microbiological results when available. Standardized infection severity grading, such as the IWGDF/IDSA diabetic foot infection severity classification, was not consistently documented across all patients, particularly because the cohort included multiple chronic wound types rather than diabetic foot ulcers alone [30,31]. Therefore, clinical infection was coded as a binary variable, indicating the presence or absence of documented clinical infection during hospitalization.

Wound pathogen results were classified according to wound exudate, deep-tissue, or other microbiological tests related to chronic wound care during hospitalization. No clear pathogen evidence was defined as no definite pathogenic microorganism detected or no definite pathogen result obtained. Single-bacterial infection was defined as the detection of only one Gram-positive or one Gram-negative bacterial species. Complex pathogen result was defined as fungal infection or mixed infection involving two or more bacterial species.

Clinical infection status and wound pathogen results were treated as distinct variables because they represented different clinical dimensions. Clinical infection reflected a clinically documented infection state, whereas wound pathogen results reflected microbiological evidence and pathogen complexity. Sepsis was not included in the complex pathogen-result category because sepsis represents a systemic clinical syndrome reflecting infection-associated organ dysfunction and host-response severity rather than microbiological complexity. Sepsis may occur in infection caused by a single microorganism, whereas polymicrobial or fungal wound infection does not necessarily result in sepsis. Therefore, incorporating sepsis into the complex pathogen-result category would combine systemic infection severity with microbiological complexity and could lead to misclassification. Accordingly, no clear pathogen evidence was not considered equivalent to the absence of clinical infection.

### 2.5. Expenditure Stratification and Analytical Framework

Patients were stratified according to percentiles of total inpatient expenditure into low-expenditure, intermediate-expenditure, high-expenditure, and very-high-expenditure groups. The low-expenditure group included patients in the 0–25th percentile, the intermediate-expenditure group included patients in the 25–75th percentile, the high-expenditure group included patients in the 75–90th percentile, and the very-high-expenditure group included patients in the 90–100th percentile. The very-high-expenditure group constituted the high-expenditure patient (HEP) cohort. Consistent with previous studies of high-cost healthcare users and inpatient diabetic foot ulcer expenditure, patients in the top 10% of total inpatient expenditure were classified as high-expenditure patients (HEPs), while the remaining patients were classified as non-HEPs [12,13,14]. HEP status was further coded as a binary outcome, with patients in the top 10% of total inpatient expenditure classified as HEPs and all other patients classified as non-HEPs.

In this study, expenditure stratification, regression analysis, and expenditure concentration analysis were used to describe different aspects of inpatient expenditure. Patients were first grouped according to total inpatient expenditure percentiles to examine how clinical characteristics and treatment patterns varied across expenditure levels and to define the HEP subgroup. Multivariable regression using log-transformed total inpatient expenditure was then performed to assess factors associated with higher expenditure in the full cohort. Finally, a Pareto-style concentration analysis was conducted to quantify the cumulative contribution of the highest-expenditure patients to total inpatient expenditure, consistent with previous high-cost-user and right-tail healthcare expenditure research [12,14,32].

### 2.6. Statistical Analysis

Continuous variables with normal distributions were presented as means ± standard deviations and were compared using *t*-tests or one-way analysis of variance, as appropriate. Non-normally distributed continuous variables were presented as medians and interquartile ranges and were compared using Mann–Whitney U tests or Kruskal–Wallis H tests, as appropriate. Categorical variables were presented as numbers and percentages and were compared using Pearson chi-square tests or Fisher exact tests. All variables included in the multivariable models were complete; therefore, all 713 patients were retained in each model, and no observations were excluded because of missing data.

Multivariable linear regression was used to examine factors associated with inpatient expenditure. Because all observed inpatient expenditure values were positive and the distribution was strongly right-skewed, total inpatient expenditure was log-transformed before model fitting, consistent with established approaches to the analysis of skewed healthcare utilization and expenditure data [33,34]. The transformation was used to improve model fit and reduce the influence of extreme expenditure values.

Length of stay was included as a hospitalization-related resource-use variable, not as a baseline clinical characteristic. Its coefficient was interpreted cautiously because length of stay may reflect underlying disease severity, treatment complexity, and postoperative recovery, while also contributing directly to inpatient expenditure through accumulated bed occupancy, nursing care, medications, consumables, diagnostic testing, and daily treatment. Surgical procedure variables were interpreted in a similar way, as indicators of resource-intensive care processes rather than purely independent baseline predictors.

Model fit and regression assumptions were assessed using several diagnostic indicators. Explanatory ability was evaluated using R^2^ and adjusted R^2^, and overall model significance was assessed using the F-statistic and corresponding p-value. Multicollinearity was examined using tolerance and variance inflation factor (VIF), with tolerance values less than 0.20 or VIF values greater than 5 considered to indicate potential multicollinearity. Standardized residual plots against standardized predicted values were used to assess heteroscedasticity and overall model fit. Residual histograms and normal probability plots were used to examine residual distribution, and standardized residuals and Cook’s distance were used to identify potentially influential observations.

Because the dependent variable in the multivariable linear regression model was log-transformed total inpatient expenditure, regression coefficients were converted into approximate percentage differences using the formula [exp(β)−1]×100%. For continuous variables, this value represents the approximate percentage difference in expenditure associated with a one-unit increase in the variable. For binary variables, it represents the approximate difference compared with the reference category or absence of the characteristic. These estimates were interpreted as differences in hospital billing expenditure, not as causal cost effects.

A reduced clinical model was also fitted as a sensitivity analysis to examine whether the main clinical associations remained after excluding downstream utilization-related variables. This model excluded length of stay and surgical procedure variables, which may partly reflect treatment intensity and disease progression during hospitalization. The full model was used to describe expenditure-associated factors after accounting for major inpatient care processes, whereas the reduced model focused on clinical characteristics before adjustment for these downstream utilization variables (Table A3).

To further examine the predefined HEP classification, multivariable logistic regression was performed with HEP status as the dependent variable. Patients in the top 10% of total inpatient expenditure were classified as HEPs, and all remaining patients were classified as non-HEPs. Owing to the small number of minor amputation cases, minor and major amputation were combined into an “amputation-related procedures” category for the HEP logistic regression analysis to improve estimation stability. Odds ratios (ORs), 95% confidence intervals (CIs), and p-values were reported.

## 3. Results

### 3.1. Patient Characteristics

The patient selection process is shown in Figure 1. After screening inpatient records identified through chronic wound-related ICD-10 codes and applying the predefined inclusion and exclusion criteria, 713 patients were included in the final analysis. The mean age was 61.98 ± 15.11 years, and 476 patients were male (66.76%). Comorbidities were common in this inpatient chronic wound cohort. Diabetes was present in 461 patients (64.66%), clinical infection in 435 patients (61.01%), peripheral arterial disease in 357 patients (50.07%), renal insufficiency in 196 patients (27.49%), and cardiovascular disease in 107 patients (15.01%). The mean mCCI was 0.45 ± 1.055, with a median of 0 (IQR: [0–0]) (Table 1). These findings indicate that inpatient chronic wound care in this tertiary hospital was largely concentrated among patients with multimorbidity, infection-related complexity, and vascular disease burden.

### 3.2. Treatment Procedures, Outcomes, and Expenditure Composition

The mean length of stay was 16.90 ± 13.46 days, with a median of 13 days (IQR: 8–21). During hospitalization, 261 patients (36.61%) received conservative treatment, 249 (34.92%) underwent debridement/drainage, 13 (1.82%) underwent minor amputation, 37 (5.19%) underwent major amputation, 140 (19.64%) underwent vascular angioplasty, and 13 (1.82%) received combined surgery. In terms of clinical outcomes, 626 patients (87.80%) improved or healed, 87 (12.20%) had unfavorable or unclear improvement, and 21 (3.00%) experienced 30-day chronic-wound-related readmission (Table 1).

The median total inpatient expenditure was CNY 16,032.78 (IQR: 9337.34–32,881.29), and the mean was CNY 26,222.81. Medical consumables accounted for the largest share of expenditure, followed by diagnostic fees, treatment fees, and medication fees (Table 2). This distribution indicates that inpatient expenditure for chronic wound care was not mainly medication-driven, but was closely related to wound-care materials, diagnostic evaluation, procedures, and other treatment-related resource use. The relatively high contribution of consumables and treatment-related fees is consistent with the need for repeated dressing changes, wound-covering materials, debridement, vascular assessment, and interventional or surgical care in patients with complex chronic wounds.

### 3.3. Factors Associated with Inpatient Expenditure and Regression Model Performance

In the multivariable linear regression model, length of stay, age, peripheral arterial disease, clinical infection, cardiovascular disease, single-bacterial result, complex pathogen result, debridement/drainage, minor amputation, major amputation, vascular angioplasty, combined surgery, and mCCI were significantly associated with higher log-transformed inpatient expenditure. Sex, diabetes mellitus, and renal insufficiency were not statistically significant after adjustment (Table 3).

The model showed good explanatory ability. The model R was 0.786, R^2^ was 0.618, and the adjusted R^2^ was 0.609, indicating that 60.9% of the variation in log-transformed inpatient expenditure was explained by the variables included in the model. The standard error of the estimate was 0.542. The overall model was statistically significant, with F = 70.332 and *p* < 0.001, and the Durbin–Watson statistic was 1.567. Multicollinearity diagnostics did not suggest substantial collinearity among the independent variables. VIF values ranged from 1.030 to 1.773, and all tolerance values were greater than 0.564. The VIF values for clinical infection status and wound pathogen-result categories were also below 5, supporting their simultaneous inclusion in the model. Visual inspection of standardized residual plots, residual histograms, and normal probability plots did not suggest major departures from regression assumptions after log-transformation of total inpatient expenditure.

To make the log-transformed regression coefficients easier to interpret, they were converted into approximate percentage differences in inpatient expenditure. Each additional hospital day was associated with approximately 3.4% higher inpatient expenditure, and each incremental year of age corresponded to a 0.4% increase in inpatient expenditure. Peripheral arterial disease and clinical infection were associated with approximately 30.9% and 46.1% higher expenditure, respectively. Compared with patients with no clear pathogen evidence, single-bacterial result and complex pathogen result were linked to roughly 14% and 43.8% greater inpatient expenditure separately. Among treatment-related variables, debridement/drainage, minor amputation, major amputation, vascular angioplasty, and combined surgery were associated with approximately 81.5%, 36.2%, 228.4%, 96.0%, and 152.9% higher expenditure in turn. Each 1-point increase in mCCI was associated with approximately 8.2% higher inpatient expenditure after multivariable adjustment. Sex, diabetes, and renal insufficiency did not exert a significant independent impact on total inpatient expenditure after multivariate adjustment. The most substantial increases in inpatient expenditure were identified in patients undergoing major amputation, combined surgery, and vascular angioplasty. Given the retrospective observational design, these estimates should be interpreted as expenditure-associated differences rather than causal cost effects.

In the reduced clinical model, age, clinical infection, renal insufficiency, cardiovascular disease, unfavorable/no clear improvement, single-bacterial result, and complex pathogen result remained significantly associated with log-transformed inpatient expenditure. In contrast, mCCI, sex, diabetes mellitus, peripheral arterial disease, and 30-day readmission were not statistically significant. No substantial multicollinearity was observed. The results are presented in Appendix A Table A3.

### 3.4. Characteristics of Expenditure Groups and High-Expenditure Patients

Patients were stratified into low-, intermediate-, high-, and very-high-expenditure groups according to percentiles of total inpatient expenditure to compare clinical characteristics and healthcare utilization across expenditure levels. The very-high-expenditure group, corresponding to the 90–100th percentile, was defined as the high-expenditure patient (HEP) group. A total of 72 patients were classified as HEPs. The mean inpatient expenditure in this subgroup was CNY 89,418.91 ± 35,555.32, and the median expenditure was CNY 72,640.27 (IQR: 65,363.21–105,210.68). Compared with the overall median inpatient expenditure of CNY 16,032.78, the median expenditure among HEPs was approximately 4.53 times higher.

Clinical complexity increased across expenditure groups. Patients in higher-expenditure groups tended to be older and had higher proportions of diabetes, peripheral arterial disease, clinical infection, and cardiovascular disease, as well as differences in wound pathogen-result distributions. They also had longer hospital stays and more frequent use of vascular angioplasty and combined surgical procedures (Table 4).

A multivariable logistic regression analysis was further performed to identify factors associated with HEP status. The results are shown in Table 5. Older age, clinical infection, cardiovascular disease, debridement/drainage, vascular angioplasty, combined surgery, and complex pathogen results were associated with higher odds of HEP status.

### 3.5. Concentration of Inpatient Expenditure

After comparing expenditure percentile groups, we examined the concentration of inpatient expenditure by ranking patients from highest to lowest total inpatient expenditure and calculating their cumulative contribution to total expenditure.

**Figure 2:**
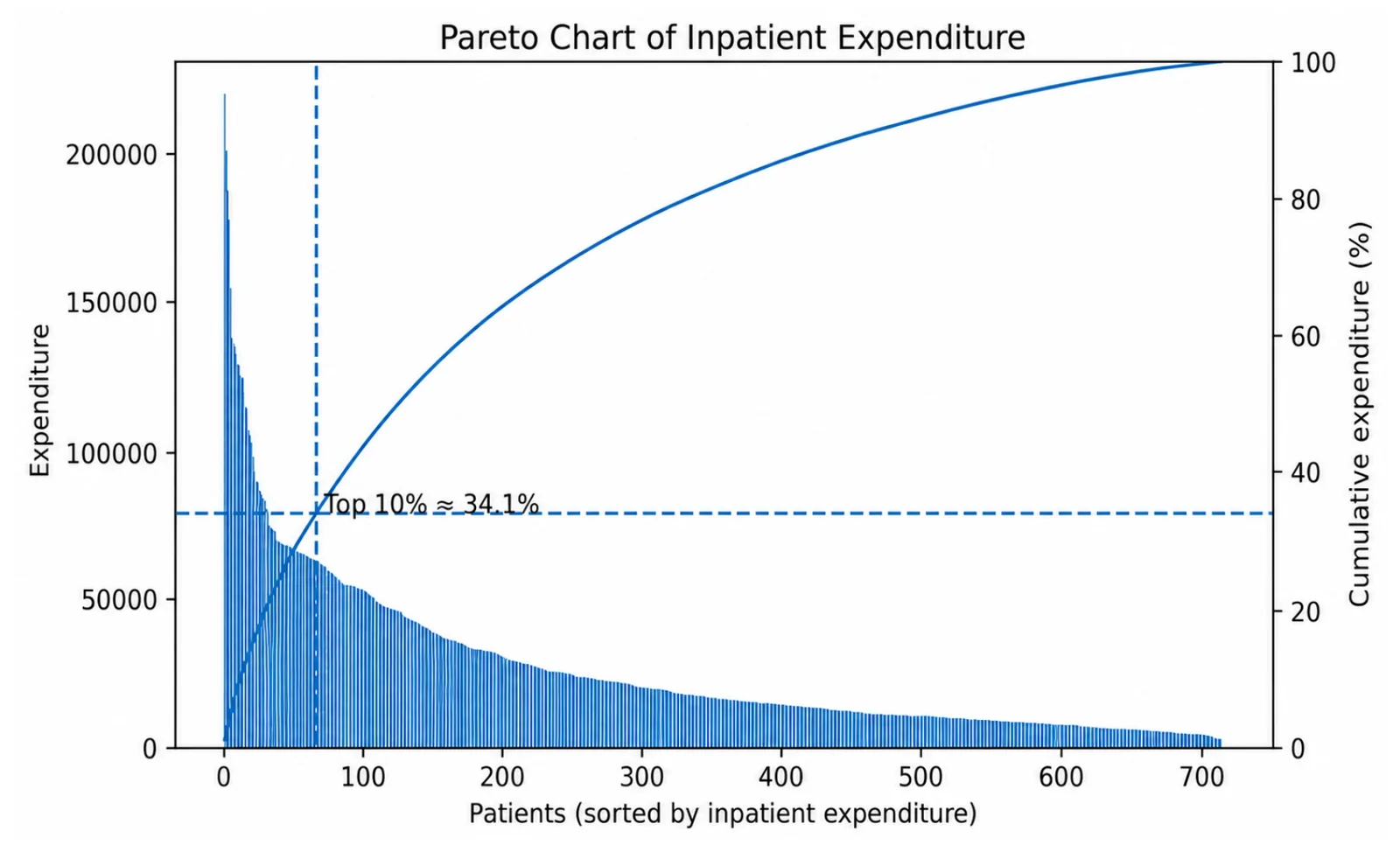
Pareto chart of inpatient expenditure. Patients are ranked from highest to lowest expenditure. The vertical dashed line indicates the top 10% of patients by expenditure, and the horizontal dashed line indicates an accumulated inpatient expenditure of approximately 34.1%.

The Pareto-style analysis showed that inpatient expenditure was clearly concentrated among a small subgroup of patients. Although HEPs accounted for only 10% of the cohort, they contributed approximately one-third of total inpatient expenditure (Figure 2). This finding indicates that resource use in inpatient chronic wound care was unevenly distributed and was disproportionately concentrated among the highest-expenditure patients.

## 4. Discussion

### 4.1. Principal Findings

Chronic wounds remain an important public health concern associated with population aging, diabetes, vascular disease, infection, and the growing burden of chronic conditions [2,35]. Previous studies have estimated the prevalence of chronic wounds in the general population at approximately 2.2 per 1000 population [1]. Chronic wounds and diabetic foot complications not only lead to long-term functional impairment and reduced quality of life but are also associated with high mortality risk and sustained health-resource use [2,36].

The different analytical approaches provided consistent and complementary evidence. The percentile-based expenditure groups showed how clinical characteristics and treatment patterns varied across the expenditure distribution. The log-transformed expenditure model identified factors associated with higher inpatient expenditure across the full cohort, while the logistic regression model further examined factors associated with membership in the predefined HEP subgroup [32]. The Pareto-style analysis then quantified the extent to which total inpatient expenditure was concentrated among the highest-expenditure patients. Together, these findings suggest that inpatient chronic wound expenditure is unevenly distributed, closely related to clinical complexity and treatment intensity, and disproportionately concentrated among a small high-expenditure subgroup [37].

### 4.2. Interpretation in Relation to Previous Literature

Our findings are consistent with previous economic studies showing that wound care and diabetic foot ulcer management impose substantial healthcare burdens. In the United Kingdom, Guest et al. estimated that the NHS managed approximately 3.8 million patients with wounds in 2017/2018, with annual wound-management costs of approximately GBP 8.3 billion, and reported that costs were substantially higher for unhealed wounds than for healed wounds [6]. Kerr et al. estimated that diabetic foot ulcers and amputations cost the NHS in England GBP 837–962 million in 2014–2015, accounting for approximately 0.8–0.9% of the NHS budget, with most expenditure related to ulceration rather than amputation [9]. In the United States, Nussbaum et al. estimated the aggregate cost of chronic wound care among Medicare beneficiaries and emphasized the policy implications of chronic non-healing wounds [38]. In the EURODIALE study, Prompers et al. showed that resource use and costs in diabetic foot ulcer care increased with disease severity, particularly when peripheral arterial disease and infection were both present [11].

Compared with these population-level or health-system-level estimates, the present study focused on inpatient expenditure at a tertiary hospital [14,39]. This difference in setting may partly explain the relatively high contribution of consumables, diagnostic services, treatment procedures, and interventional care observed in our cohort. Recent inpatient diabetic foot ulcer research in an Asian multiethnic population also showed that high-cost users contributed disproportionately to total inpatient expenditure and were characterized by greater comorbidity burden and more complex treatment needs [14]. Therefore, our finding that the top 10% of patients accounted for approximately one-third of total inpatient expenditure is broadly consistent with the international pattern of expenditure concentration, although the exact composition of expenditure may differ according to care setting, payment mechanism, service organization, and patient case mix.

### 4.3. Clinical and Economic Implications

In terms of expenditure composition, medical consumables accounted for the largest proportion of inpatient expenditure (37.01%), followed by diagnostic fees, treatment fees, and medication fees. This pattern is consistent with the clinical characteristics of inpatient chronic wound management. Patients with chronic wounds often require repeated dressing changes, wound-covering materials, disposable treatment consumables, surgical consumables, and, in some cases, vascular interventional materials. For patients with infection, ischemia, or necrotic tissue, repeated debridement, negative-pressure wound therapy, revascularization, or amputation may also be required. Therefore, expenditure management in chronic wound care should not focus only on medication fees or length of stay, but should also consider standardized use of consumables, appropriate treatment indications, and optimization of inpatient care pathways. Previous studies on the economic burden of wounds have similarly shown that wound-management expenditure includes not only inpatient services, but also dressings, nursing care, outpatient follow-up, long-term care, and productivity losses [6,40].

Regarding treatment modalities, vascular angioplasty, combined surgery, major amputation, and debridement/drainage were significantly associated with higher inpatient expenditure. Minor amputation also showed a positive association with inpatient expenditure (β = 0.309, 95% CI: 0.002–0.616; *p* = 0.048), corresponding to approximately 36.2% higher expenditure. Given the borderline statistical significance and the small number of minor amputation cases, this finding should be interpreted cautiously [3,41,42]. Vascular angioplasty and combined surgery showed relatively large regression coefficients, suggesting that patients requiring complex or staged treatment pathways had substantially greater resource use. Debridement/drainage often reflects infection, abscess formation, or necrotic tissue burden, whereas major amputation usually indicates advanced tissue necrosis, uncontrolled infection, or severe ischemia; and combined surgery suggests that multiple interventions are required either during the same admission or in stages. These procedures are commonly accompanied by greater use of operating-room resources, anesthesia, consumables, nursing care, postoperative monitoring, and wound management. Surgical intervention should therefore be interpreted not only as a treatment modality but also as an indirect marker of disease complexity and resource-intensive care.

Comorbidity burden was also associated with inpatient expenditure. Although diabetes was not statistically significant after multivariable adjustment, it may still contribute indirectly to resource use through its association with peripheral arterial disease, infection, wound pathogen results, revascularization, and surgical intervention. In the multivariable analysis, peripheral arterial disease, clinical infection, cardiovascular disease, and higher mCCI were significantly associated with higher inpatient expenditure [14,43]. These conditions may reflect greater disease complexity, delayed wound repair, increased diagnostic and treatment requirements, and longer hospitalization. Given the retrospective observational design, these regression results should be interpreted as statistical associations rather than causal effects.

Wound pathogen results were also associated with inpatient expenditure. Both single-bacterial result and complex pathogen result were associated with higher expenditure, with a larger regression coefficient observed for complex pathogen result. In this study, complex pathogen result included fungal infection or mixed infection involving two or more bacterial species. This category may reflect a more disturbed wound microenvironment, greater pathogen complexity, or higher microbial burden. Sepsis was not incorporated into this microbiological category because it reflects systemic infection severity and host-response dysfunction rather than pathogen complexity. Patients in the complex pathogen-result category may require more frequent microbiological testing, antimicrobial susceptibility testing, empirical or combination antimicrobial therapy, repeated debridement, negative-pressure wound therapy, and longer inpatient observation. Previous studies have suggested that biofilms and fungal infection in chronic wounds may delay healing and increase treatment difficulty [44,45]. The IWGDF/IDSA guideline emphasizes that wound infection should be diagnosed based on local or systemic inflammatory manifestations and assessed according to infection severity. Antimicrobial selection should consider likely or confirmed pathogens, susceptibility results, infection severity, adverse events, drug interactions, availability, and cost. When bone infection is suspected, bone specimens should be obtained for culture when necessary [29]. Therefore, early microbiological assessment and infection stratification may help reduce prolonged empirical therapy, inappropriate antimicrobial use, and repeated ineffective interventions in patients with suspected complex pathogen result.

Peripheral arterial disease was also associated with higher inpatient expenditure. In patients with chronic wounds, impaired tissue perfusion can delay granulation and epithelialization and increase the risk of necrosis, infection, amputation, and the need for revascularization [11,46]. In this study, vascular angioplasty showed the largest regression coefficient among the significant variables, indicating that revascularization-related care was closely linked to higher inpatient resource use. Although angioplasty may improve local perfusion and support wound repair, it often requires vascular imaging, interventional consumables, perioperative management, and post-procedure monitoring. These processes are resource-intensive and may partly explain the high expenditure associated with angioplasty. Early perfusion assessment, clear evaluation of revascularization indications, and avoidance of unnecessary delays in patients at risk of ischemia may help improve treatment efficiency.

The regression results should be interpreted as associations rather than causal effects. Length of stay and surgical procedures may represent downstream care processes that partly reflect disease severity [47], infection burden, ischemia, comorbidity, and treatment complexity. Their inclusion in the full model may therefore attenuate the total associations between upstream clinical conditions and inpatient expenditure. Nevertheless, the full model was retained because it reflects hospital-level resource use and helps identify major clinical and utilization-related factors associated with inpatient expenditure. To address potential overadjustment, we also performed a reduced clinical model excluding length of stay and surgical procedure variables. The reduced model focused on upstream clinical characteristics before adjustment for downstream utilization, whereas the full model described expenditure-associated factors after accounting for major inpatient care processes.

Potential bidirectional relationships should also be considered. Infection, peripheral arterial disease, and comorbidity may increase the need for prolonged hospitalization, vascular assessment, debridement, amputation, or combined surgery, which may in turn contribute to higher inpatient expenditure [29,48]. Conversely, patients undergoing intensive diagnostic and therapeutic procedures may remain hospitalized longer because of perioperative monitoring, staged wound management, or post-procedure recovery. Thus, clinical severity, treatment intensity, length of stay, and expenditure are closely interconnected in inpatient chronic wound care. The present retrospective analysis could not fully separate these pathways, and the regression coefficients should therefore be interpreted as indicators of resource use rather than independent causal effects.

Length of stay was strongly associated with higher inpatient expenditure, but this association requires cautious interpretation because length of stay has a dual role in inpatient expenditure analysis. Prolonged hospitalization may reflect underlying disease severity and treatment complexity, including delayed wound healing, difficult infection control, ischemia requiring perfusion assessment, repeated procedures, and management of complications [42,49]. At the same time, length of stay is also part of the expenditure-generating process, as bed occupancy, nursing care, daily treatment, medications, consumables, diagnostic testing, and monitoring accumulate during hospitalization. Therefore, length of stay should be interpreted as a resource-use indicator that captures disease severity, treatment complexity, and cumulative inpatient care, rather than as a completely independent causal determinant of expenditure. Early risk stratification, timely infection and perfusion assessment, and coordinated multidisciplinary decision-making may help reduce ineffective waiting and repeated interventions.

The magnitude of the regression coefficients also provides clinically meaningful information about resource use. In the full model, vascular angioplasty, combined surgery, major amputation, debridement/drainage, clinical infection, and complex pathogen result showed relatively large positive associations with log-transformed inpatient expenditure [29,42,50]. These variables should not be viewed as isolated predictors; rather, they represent clinically severe and resource-intensive care pathways. Peripheral arterial disease and vascular angioplasty reflect ischemia-related wound complexity, vascular imaging, interventional materials, perioperative monitoring, and delayed wound repair. Clinical infection and wound pathogen results indicate higher infection burden, microbiological testing, antimicrobial therapy, repeated debridement, and longer inpatient observation. Major amputation and combined surgery suggest advanced tissue necrosis, uncontrolled infection, severe ischemia, or multiple staged procedures. Taken together, these findings indicate that higher expenditure was concentrated mainly among patients requiring complex pathogen result control, perfusion restoration, and surgical management, rather than among patients receiving routine wound care alone.

### 4.4. Health-System and Policy Implications

Although this study focused mainly on patient-level clinical and treatment-related factors, inpatient expenditure is also shaped by system-level and institutional factors. In China, hospital-centered service delivery, insurance reimbursement arrangements, regional variation in medical resources, hospital pricing policies, and ongoing DRG/DIP-related payment reforms may all influence inpatient expenditure and cost composition [15,17,19]. Under case-based payment reforms, hospitals face stronger incentives to improve efficiency, reduce unnecessary length of stay, standardize consumable use, and optimize clinical pathways [19,21]. However, patients with chronic wounds often require individualized and multidisciplinary care, especially when infection, ischemia, multimorbidity, or complex surgery is present. Therefore, expenditure management strategies should avoid simple cost compression and should instead emphasize appropriate early assessment, standardized treatment pathways, rational use of consumables, timely vascular evaluation, and coordinated multidisciplinary care [50]. The lack of detailed insurance reimbursement data, out-of-pocket payment information, and institutional pricing variables in this study limited our ability to separate patient-level clinical factors from health-system and hospital-level influences on expenditure.

The identification of HEPs further highlights the importance of targeted resource management in inpatient chronic wound care. Although these 72 patients accounted for only 10% of the cohort, they contributed approximately 34.1% of total inpatient expenditure, showing a marked concentration of resource use [12]. This finding is consistent with the international pattern that a small proportion of patients consume a disproportionate share of healthcare resources [12,13,51]. Wodchis et al. found that high-cost users represented a small proportion of the population but had highly concentrated and persistent health-resource use. Holle et al. similarly reported that healthcare expenditures in the United States remained concentrated among a small group of high spenders [13]. In the present study, HEPs were older and had higher proportions of diabetes, peripheral arterial disease, clinical infection, complex pathogen results, and cardiovascular disease. They also underwent vascular angioplasty and combined surgery more frequently. These findings suggest that HEPs were not simply patients with higher expenditure, but a clinically high-risk subgroup characterized by older age, infection-related complexity, impaired perfusion, complex pathogen results, and intensive treatment needs [14].

The identification of HEPs has implications beyond describing expenditure concentration. In this study, HEPs appeared to represent a clinically identifiable high-risk subgroup characterized by multimorbidity, infection burden, impaired perfusion, and complex treatment needs. Early identification of patients at risk of becoming HEPs may help hospitals implement targeted case management, early vascular assessment, timely infection stratification, rational antimicrobial use, nutritional and glycemic optimization, and coordinated surgical decision-making [52]. It may also support more efficient allocation of multidisciplinary wound-care resources to patients most likely to require intensive inpatient care pathways.

These findings suggest that expenditure management in inpatient chronic wound care should shift from simple cost compression to targeted management of high-risk patients. Older patients and those with greater comorbidity burden, peripheral arterial disease, cardiovascular disease, clinical infection, complex pathogen results [31], or an anticipated need for vascular or surgical intervention may benefit from early risk stratification and coordinated care [53,54]. Potential hospital-level strategies include early wound and infection assessment, vascular screening, glycemic and nutritional optimization, antimicrobial stewardship, standardized selection of wound-care materials, and timely multidisciplinary consultation. However, these strategies should be regarded as potential approaches to resource optimization rather than proven cost-reduction interventions, given the observational design of this study.

The practical implication of these findings is that patients at risk of high inpatient expenditure may be partly identifiable early during hospitalization. Patients with peripheral arterial disease, clinical infection, wound pathogen results, high comorbidity burden, or anticipated need for vascular or surgical intervention may benefit from early risk stratification and targeted care coordination [16,55,56]. At the hospital level, this may include early wound assessment, infection evaluation, vascular screening, glycemic and nutritional optimization, antimicrobial stewardship, standardized selection of wound-care materials, and timely multidisciplinary consultation involving wound-care specialists, vascular surgery, endocrinology, infectious disease, nursing, rehabilitation, and discharge-planning teams.

These strategies should not be interpreted as proven cost-reduction interventions based on the present observational data. Rather, they represent potential approaches to resource optimization suggested by the expenditure-associated patterns observed in this study. Future prospective studies are needed to evaluate whether early triage, standardized care pathways, multidisciplinary case management, and post-discharge follow-up can reduce avoidable high-expenditure admissions while maintaining or improving wound-healing outcomes [30,54,55,56].

### 4.5. Limitations and Future Research Directions

This study has several limitations. First, it was a single-center retrospective study based on discharge abstract data from a tertiary hospital in the Inner Mongolia Autonomous Region of China. Patients treated in tertiary hospitals typically present with more severe wounds, higher comorbidity burden, more frequent infection or ischemia, and greater need for surgical or interventional treatment compared with those managed in lower-level hospitals, outpatient wound clinics, or community care settings. In addition, cost structures, reimbursement arrangements, hospital pricing policies, resource availability, and treatment patterns may vary across regions, hospital levels, and healthcare systems. These factors may limit the generalizability of the observed expenditure levels, expenditure composition, and characteristics of high-expenditure patients (HEPs) to other settings.

Second, potential selection bias should be considered. The study included only hospitalized patients with valid chronic wound-related diagnostic and expenditure records and excluded admissions primarily unrelated to wound treatment. While this approach improved cohort consistency, it may have excluded patients with milder chronic wounds managed in outpatient or community settings, as well as complex multimorbid patients whose principal reason for admission was another acute condition but who still required wound-related care. As a result, the study may overrepresent more severe inpatient chronic wound cases and may underestimate wound-related expenditure among patients admitted primarily for other conditions.

Third, the retrospective observational design limited causal inference and may have introduced information bias and residual confounding. Although multivariable regression was used to adjust for measured covariates, several clinically important variables were not consistently available, including wound area, wound depth, wound duration, ischemia severity, standardized infection severity grading, nutritional status, functional status, frailty, pain severity, perfusion imaging results, and detailed wound classification. The absence of these variables limited the depth of clinical interpretation and may have influenced the estimated associations between clinical characteristics and inpatient expenditure.

Fourth, potential endogeneity, overadjustment, and reverse causation should be considered. Some variables included in the regression model, particularly length of stay and surgical procedures, may have represented downstream consequences of disease severity rather than independent baseline exposures. Length of stay may reflect disease severity, treatment complexity, and postoperative recovery, while also contributing directly to expenditure accumulation through bed occupancy, nursing care, daily treatment, medications, consumables, diagnostic testing, and monitoring. Similarly, surgical procedures may indicate severe infection, ischemia, necrosis, or staged wound management, while also being associated with greater resource use. Although a reduced clinical model excluding length of stay and surgical procedure variables was performed to reduce potential overadjustment by downstream utilization-related factors, the retrospective design could not fully separate causal pathways, mediation effects, or bidirectional relationships. Therefore, the regression coefficients should be interpreted as expenditure-associated differences rather than independent causal effects.

Fifth, the interpretation of economic data is limited by the nature of hospital billing records. The expenditure data used in this study primarily reflect inpatient charges recorded during hospitalization and may not correspond to the hospital’s true economic costs, insurance reimbursement amounts, or patients’ out-of-pocket payments. In addition, this study focused on direct inpatient expenditure and did not capture outpatient care, post-discharge wound management, rehabilitation, recurrence-related care, productivity loss, transportation costs, or informal family caregiving expenses. Therefore, the findings should be interpreted as patterns of inpatient hospital billing expenditure rather than a comprehensive estimate of the overall economic burden of chronic wounds from patient, payer, or societal perspectives.

Sixth, the ICD-10 code list was used as a broad screening tool rather than as a validated stand-alone diagnostic algorithm. Although all screen-positive records underwent medical-record adjudication, records that did not meet the initial code-based screen were not independently reviewed. Therefore, the sensitivity, specificity, and negative predictive value of the screening procedure could not be estimated, and some eligible cases may have been missed. In addition, the locally implemented ICD-10 extensions and hospital coding practices may limit the generalizability of the algorithm to other institutions.

Finally, HEPs were defined as patients in the top 10% of total inpatient expenditure, which is a commonly used but threshold-dependent classification. Although multivariable logistic regression was performed to examine factors associated with HEP status, alternative thresholds may lead to different classifications of high-expenditure patients. Future studies should explore alternative HEP definitions, develop and externally validate prediction models for high-expenditure risk, and evaluate whether early triage, standardized care pathways, multidisciplinary case management, and post-discharge follow-up can optimize resource use while maintaining or improving wound-healing outcomes. Multicenter prospective studies incorporating detailed wound-severity indicators, standardized infection grading, hospital cost-accounting data, insurance reimbursement information, patient out-of-pocket payments, outpatient and post-discharge expenditure, and patient-centered outcomes are needed to provide a more comprehensive understanding of the economic burden of chronic wounds.

## 5. Conclusions

In this single-center retrospective study, inpatient treatment for chronic wounds was associated with substantial expenditure and clinical complexity. Expenditure was mainly concentrated in medical consumables, diagnostic services, treatment procedures, and medications, indicating that inpatient chronic wound care involves not only pharmacological treatment but also wound-care materials, diagnostic evaluation, vascular assessment, and surgical or interventional management. Length of stay, comorbidity burden, clinical infection, wound pathogen results, peripheral arterial disease, cardiovascular disease, and treatment procedures were associated with higher inpatient expenditure. These factors should be interpreted as indicators of clinical complexity and resource-intensive care rather than independent causal determinants.

The identification of HEPs provides additional practical value. Although HEPs represented a small proportion of the cohort, they contributed substantially to total inpatient expenditure and showed greater infection-related complexity, impaired perfusion, and more intensive treatment needs. These findings suggest that high-expenditure chronic wound care may be better addressed through patients at risk of high expenditure being partly identified during hospitalization, multidisciplinary case management, timely infection and vascular assessment, standardized use of consumables, and coordinated surgical decision-making rather than simple cost reduction alone. By combining expenditure stratification, continuous expenditure modeling, and expenditure concentration analysis, this study provides a hospital-level framework for understanding not only which factors are associated with higher inpatient expenditure but also how expenditure is distributed and concentrated among patients with chronic wounds.

Future studies should incorporate hospital cost-accounting data, insurance reimbursement information, patient out-of-pocket payments, outpatient and post-discharge costs, and indirect costs to provide a more comprehensive assessment of the economic burden of chronic wounds.

## Figures and Tables

**Table 1:** Demographic characteristics, comorbidities, treatments, and clinical outcomes of the study population (N = 713).

Variable	Value, n (%) or Mean ± SD
Demographics	
Age, years	61.98 ± 15.106
Male sex	476 (66.76%)
Female sex	237 (33.24%)
Diabetes	461 (64.66%)
Peripheral arterial disease	357 (50.07%)
Clinical infection	435 (61.01%)
Renal insufficiency	196 (27.49%)
Cardiovascular disease	107 (15.01%)
Wound pathogen results	
No clear pathogen evidence	435 (61.01%)
Single-bacterial result	214 (30.01%)
Complex pathogen result	64 (8.98%)
Treatments during admission	
Conservative treatment	261 (36.61%)
Debridement/drainage	249 (34.92%)
Minor amputation	13 (1.82%)
Major amputation	37 (5.19%)
Vascular angioplasty	140 (19.64%)
Combined surgery	13 (1.82%)
Clinical outcomes	
Unfavorable/no clear improvement	87 (12.20%)
Improvement or healing	626 (87.80%)
30-day chronic wound-related readmission	21 (3.00%)
Length of stay during admission:	
Mean (SD), days	16.90 (13.458)
Median (IQR), days	13 (8–21)
Modified Charlson Comorbidity Index (mCCI):	
Mean (SD)	0.45 (1.055)
Median (IQR)	0 (0–0)

Note: Data are presented as n/N (%), mean ± standard deviation, or median (interquartile range), as appropriate. Single-bacterial result was defined as detection of only one Gram-positive or one Gram-negative bacterium; complex pathogen result was defined as fungal infection or mixed infection involving two or more bacterial species. Denominators are reported separately when data are missing. mCCI, modified Charlson Comorbidity Index; SD, standard deviation; IQR, interquartile range.

**Table 2:** Expenditure per patient and major expenditure components (N = 713).

Item	Value
Expenditure per patient	
Mean (SD), CNY	26,222.81 (27,368.38)
Median (IQR), CNY	16,032.78 (9337.34–32,881.29)
Expenditure components per patient	
Comprehensive medical service fees	7.82%
Diagnostic fees	20.45%
Treatment fees	20.23%
Medication fees	13.37%
Blood and blood-product fees	0.49%
Medical consumable fees	37.01%
Other fees	0.62%

Note: Expenditure values are reported in Chinese yuan (CNY) and were derived from itemized inpatient billing records. They represent hospital charges recorded during the index hospitalization rather than true inpatient expenditure, insurance reimbursement, or patient out-of-pocket expenditure. Component percentages were calculated relative to total inpatient expenditure.

**Table 3:** Full multivariable linear regression analysis of factors associated with total inpatient expenditure (analytic N = 713).

Variable	β	95% CI	Approximate Percentage Difference, %	*p*-Value
Length of stay, days	0.033	0.030 to 0.036	3.4	<0.001
Age	0.004	0.001 to 0.007	0.4	0.010
Sex	0.032	−0.054 to 0.118	3.3	0.468
Diabetes	0.082	−0.027 to 0.191	8.5	0.139
Peripheral arterial disease	0.269	0.163 to 0.375	30.9	<0.001
Clinical infection	0.379	0.292 to 0.467	46.1	<0.001
Renal insufficiency	−0.001	−0.107 to 0.105	−0.1	0.980
Cardiovascular disease	0.232	0.112 to 0.353	26.1	<0.001
Single-bacterial result	0.131	0.036 to 0.226	14.0	0.007
Complex pathogen result	0.363	0.214 to 0.512	43.8	<0.001
Debridement/drainage	0.596	0.490 to 0.702	81.5	<0.001
Minor amputation	0.309	0.002 to 0.616	36.2	0.048
Major amputation	1.188	1.067 to 1.309	228.4	<0.001
Vascular angioplasty	0.673	0.477 to 0.870	96.0	<0.001
Combined surgery	0.928	0.616 to 1.240	152.9	<0.001
mCCI	0.079	0.039 to 0.118	8.2	<0.001

Note: The full multivariable model included length of stay, age, sex, diabetes mellitus, peripheral arterial disease, clinical infection, renal insufficiency, cardiovascular disease, wound pathogen-result categories, treatment procedures during admission, and the modified charlson comorbidity index (mCCI). No clear pathogen evidence was used as the reference category for wound pathogen results, and conservative treatment/no surgery was used as the reference category for treatment procedures. Regression coefficients (β) were estimated using log-transformed total inpatient expenditure. Approximate percentage differences were calculated as [exp(β)−1] × 100%. mCCI was entered as a continuous variable. CI, confidence interval; mCCI, modified charlson comorbidity index. Model fit statistics for this multivariable linear regression: $R^{2}=0.618$, adjusted $R^{2}=0.609$, standard error of the estimate = 0.542, Durbin-Watson statistic = 1.567. The overall *F* test of the model was statistically significant (F=70.332, p<0.001).

**Table 4:** (**a**) Demographic characteristics, comorbidities, and wound pathogen results across expenditure groups (N = 713). (**b**) Treatment procedures, healthcare utilization indicators, and clinical outcomes across expenditure groups (N = 713). (**c**) Inpatient expenditure distribution across expenditure groups (N = 713).

Variable	Very High (n = 72)	High (n = 106)	Intermediate (n = 356)	Low (n = 179)	*p*-Value
(**a**)					
Demographic characteristics and comorbidities:					
Age, years	68.76 ± 12.70	66.1 ± 13.83	61.12 ± 13.93	58.51 ± 17.50	<0.001
mCCI, median (IQR)	0.75 (0.00–2.00)	0.00 (0.00–0.00)	0.00 (0.00–0.00)	0.00 (0.00–0.00)	0.686
Male sex	40 (55.6%)	71 (67.0%)	248 (69.7%)	117 (65.4%)	0.134
Female sex	32 (44.4%)	35 (33.0%)	108 (30.3%)	62 (34.6%)	
Diabetes	55 (76.4%)	75 (70.8%)	223 (62.6%)	108 (60.3%)	0.043
Peripheral arterial disease	48 (66.7%)	55 (51.9%)	172 (48.3%)	82(45.8%)	0.020
Clinical infection	56 (77.8%)	79 (74.5%)	213 (59.8%)	87 (48.6%)	<0.001
Renal insufficiency	22 (30.6%)	22 (20.8%)	100 (28.1%)	52 (29.1%)	0.386
Cardiovascular disease	22 (30.6%)	22 (20.8%)	43 (12.1%)	20 (11.2%)	<0.001
Wound pathogen results:					
No clear pathogen evidence	46 (63.9%)	43 (40.6%)	207 (58.2%)	139 (77.7%)	<0.001
Single-bacterial result	11 (15.3%)	47 (44.3%)	123 (34.6%)	33 (18.4%)	
Complex pathogen result	15 (20.8%)	16 (15.1%)	26 (7.3%)	7 (3.9%)	
(**b**)					
Treatment procedures during admission:					
No surgery	6 (8.3%)	18 (17.0%)	117 (32.9%)	120 (67.0%)	<0.001
Debridement/drainage	21 (29.2%)	46 (43.4%)	148 (41.6%)	34 (19.0%)	
Minor amputation	0 (0%)	3(2.8%)	6 (1.7%)	4 (2.2%)	
Major amputation	3 (4.2%)	7(6.6%)	27 (7.6%)	0 (0%)	
Vascular angioplasty	36 (50.0%)	26 (24.5%)	57 (16.0%)	21 (11.7%)	
Combined surgery	6 (8.3%)	6 (5.7%)	1 (0.3%)	0 (0%)	
Length of stay after admission:					
Mean (SD), days	27.19 ± 21.49	27.34 ± 16.83	16.30 ± 9.20	7.75 ± 3.44	<0.001
Median (IQR), days	21.5 (11.5–34)	24 (15–37)	14 (10–20)	7 (5–10)	
Outcomes:					
Improvement or healing	63 (87.5%)	91 (85.8%)	318 (89.3%)	154 (86.0%)	0.642
Unfavorable/no clear improvement	9 (12.5%)	15 (14.2%)	38 (10.7%)	25 (14.0%)	
30-day chronic-wound-related readmission	2 (2.8%)	2 (1.9%)	13 (3.7%)	4 (2.2%)	0.715
(**c**)					
Total inpatient expenditure:					
Mean (SD)	89,418.91	45,217.00	17,844.42	6616.11	<0.001
CNY	(35,555.32)	(7932.46)	(6609.74)	(1886.11)	
Median (IQR)	72,640.27	45,249.87	16,032.78	6665.34	
CNY	(65,363.21–105,210.68)	(37,976.8–52,848.9)	(11,944.71–22,289.96)	(5128.43–8367.37)	

Note: The very high group comprised patients in the 90th–100th expenditure percentiles, the high group comprised the 75th–<90th percentiles, the intermediate group comprised the 25th–<75th percentiles, and the low group comprised the lowest 25th percentiles. The very high group was designated as the high-expenditure patient group. Data are presented as n/N (%), mean ± standard deviation, or median (interquartile range), as appropriate.

**Table 5:** Multivariable logistic regression analysis of factors associated with high-expenditure patient status (analytic N = 713; HEPs, n = 72; non-HEPs, n = 641).

Variable	OR	95% CI	*p*-Value
Age, years	1.048	1.019–1.078	<0.001
Male sex	1.585	0.876–2.868	0.128
Modified Charlson Comorbidity Index (mCCI)	1.148	0.849–1.552	0.369
Diabetes	1.819	0.815–4.063	0.144
Peripheral arterial disease	1.141	0.527–2.472	0.737
Clinical infection	3.662	1.792–7.484	<0.001
Renal insufficiency	1.021	0.488–2.139	0.956
Cardiovascular disease	2.443	1.150–5.190	0.020
30-day chronic-wound-related readmission	1.278	0.243–6.714	0.772
Unfavorable/no clear improvement	0.998	0.404–2.468	0.997
Treatment procedures during admission:			
Debridement/drainage vs reference	9.111	3.094–26.835	<0.001
Amputation-related procedures vs reference	3.918	0.812–18.909	0.089
Vascular angioplasty vs reference	44.913	14.897–135.409	<0.001
Combined surgery vs reference	70.445	13.923–356.416	<0.001
Wound pathogen results:			
Single-bacterial result vs reference	0.508	0.227–1.141	0.101
Complex pathogen result vs reference	3.735	1.594–8.749	0.002

Note: HEP status was defined as membership in the top 10% of total inpatient expenditure. HEP = 1, non-HEP = 0; ORs and 95% CIs were estimated using multivariable logistic regression. Categorical predictors were entered as dummy variables. The reference category for wound pathogen results was no clear pathogen evidence, and the reference category for treatment was conservative treatment/no surgery. OR, odds ratio; CI, confidence interval; HEP, high-expenditure patient. Minor and major amputations were combined as “amputation-related procedures” because of the small number of minor amputation cases.

## Data Availability

The data supporting the findings of this study are available from the corresponding author upon reasonable request. The data are not publicly available because they contain potentially identifiable and sensitive patient information and are subject to institutional and ethical restrictions.

## Abbreviations

The following abbreviations are used in this manuscript:

mCCI	modified Charlson Comorbidity Index
CNY	Chinese Yuan
HEPs	High-Expenditure Patients
ICD	International Classification of Diseases
IDSA	Infectious Diseases Society of America
IQR	Interquartile Range
IWGDF	International Working Group on the Diabetic Foot
LOS	Length of Stay
NHS	National Health Service
PVD	Peripheral Vascular Disease
SD	Standard Deviation
USD	United States Dollar

## Appendix A

**Table A1:** ICD-10 codes used for candidate chronic wound case screening.

Wound-Related Category	ICD/Diagnostic Label	ICD Code	Used for Candidate Screening	Sufficient Alone for Confirmation	Required Clinical Confirmation
Diabetes-related wound	Type 2 diabetes mellitus with peripheral vascular disease and gangrene	E11.36xx01	Yes	No	Diabetes plus explicit foot ulcer
	Type 1 diabetes mellitus with peripheral vascular disease and gangrene	E11.59xx21	Yes	No	Diabetes plus explicit foot ulcer
	Type 2 diabetes mellitus with acute skin gangrene	E11.59xx45	Yes	No	Diabetes plus explicit foot ulcer
	Type 2 diabetes mellitus with lower limb ulcer	E11.59xx13	Yes	No	Diabetes plus explicit foot ulcer
	Type 2 diabetes mellitus with foot ulcer	E11.89xx00	Yes	No	Diabetes plus explicit foot ulcer
	Type 2 diabetes mellitus with lower limb infection	E11.59xx31	Yes	No	Diabetes plus explicit foot ulcer
	Type 2 diabetes mellitus with peripheral vascular disease, foot ulcer, and gangrene	E11.59xx72	Yes	No	Diabetes plus explicit foot ulcer
	Type 2 diabetes mellitus with foot disease	E11.53	Yes	No	Diabetes plus explicit foot ulcer
	Type 2 diabetes mellitus with ulcer	E11.54	Yes	No	Diabetes plus explicit foot ulcer
	Type 2 diabetes mellitus with gangrene	E11.59	Yes	No	Diabetes plus explicit foot ulcer
	Type 2 diabetes mellitus with peripheral vascular disease and gangrene	E11.52xx79	Yes	No	Diabetes plus explicit foot ulcer
	Type 2 diabetes mellitus with foot ulcer and peripheral vascular disease	E11.79xx01	Yes	No	Diabetes plus explicit foot ulcer
	Type 2 diabetes mellitus with toe ulcer and peripheral neuropathy	E11.79xx02	Yes	No	Diabetes plus explicit foot ulcer
	Diabetes mellitus with foot gangrene	E11.94xx41	Yes	No	Diabetes plus explicit foot ulcer
	Diabetes mellitus with foot disease	E14.31xx95	Yes	No	Diabetes plus explicit foot ulcer
	Diabetes mellitus with lower-limb infection and gangrene	E14.98xx51	Yes	No	Diabetes plus explicit foot ulcer
	Type 1 diabetes mellitus with foot ulcer and peripheral vascular disease	E10.79xx21	Yes	No	Diabetes plus explicit foot ulcer
	Type 1 diabetes mellitus ulcer	E10.518	Yes	No	Persistent abnormality identifiable wound
	Type 2 diabetes mellitus ulcer	E11.384	Yes	No	Persistent abnormality identifiable wound
	Type 2 diabetes gangrene	E11.59	Yes	No	Persistent abnormality identifiable wound
Arterial wound	Atherosclerotic gangrene of the lower extremities	I70.261	Yes	No	Persistent abnormality identifiable wound
	Atherosclerosis, extensive disease of the lower extremities with gangrene	I70.26xx72	Yes	No	Persistent abnormality identifiable wound
	Obliterating arteritis with gangrene	I70.361xx01	Yes	No	Persistent abnormality identifiable wound
	Arterial occlusion of the right lower extremity with foot gangrene	I74.3Bxx06	Yes	No	Persistent abnormality identifiable wound
Venous wound	Varicose veins of both lower extremities with ulcer	I83.40xx100	Yes	Yes	Site, wound documentation and duration
	Varicose vein of the left lower extremity with ulcer	I83.810	Yes	Yes	Site, wound documentation and duration
	Varicose veins of both lower extremities with ulcer	I83.40xx100	Yes	Yes	Site, wound documentation and duration
	Varicose vein of the left lower extremity with ulcer	I83.810	Yes	Yes	Site, wound documentation and duration
Infection wound	Necrosis of the left lower leg with infection	L08.99	Yes	No	Wound plus clinical infection evidence
	Infection of the right foot	B99.8xx01	Yes	No	Wound plus clinical infection evidence
	Infection of the skin of the buttocks	L08.91xx01	Yes	No	Wound plus clinical infection evidence
	Infection of the skin	L08.90xx03	Yes	No	Wound plus clinical infection evidence
	Infected ulcer next to the right heel	L08.91xx04	Yes	No	Wound plus clinical infection evidence
	Infection of the hand	L08.90xx17	Yes	No	Wound plus clinical infection evidence
	Serious infection of the lower extremity	L08.90xx27	Yes	No	Wound plus clinical infection evidence
	Infection of the toe	L08.90xx22	Yes	No	Wound plus clinical infection evidence
	Infection of the scalp	L08.90xx13	Yes	No	Wound plus clinical infection evidence
	Infection of the leg	L08.90xx21	Yes	No	Wound plus clinical infection evidence
	Infected sinus tract of the skin	L08.501	Yes	No	Wound plus clinical infection evidence
	Infection of scar tissue	L90.5	Yes	No	Wound plus clinical infection evidence
	Infection of the skin of the trunk after radiotherapy-related breakdown	L08.902	Yes	No	Wound plus clinical infection evidence
	Infection of the skin of the lower extremity	L08.904	Yes	No	Wound plus clinical infection evidence
	Soft tissue infection of the neck	L08.998	Yes	No	Wound plus clinical infection evidence
	Soft tissue infection of the abdominal wall	L08.917	Yes	No	Wound plus clinical infection evidence
	Soft tissue infection of the left foot	L08.913	Yes	No	Wound plus clinical infection evidence
Pressure wound	Stage III pressure ulcer in a pressure area	L89.243	Yes	Yes	Site, wound documentation and duration
	Multiple stage IV pressure ulcers with infection	L89.31xx08	Yes	Yes	Site, wound documentation and duration
	Pressure ulcer	L89.xx001	Yes	Yes	Site, wound documentation and duration
	Ulcer of the lower extremity, unspecified classification	L97.909	Yes	Yes	Site, wound documentation and duration
Iatrogenic wound	Poor healing of postoperative incision	T81.89xx17	Yes	No	Persistent wound and wound-related care
	Infection of the knee joint after surgery for left patellar fracture	T81.445	Yes	No	Persistent wound and wound-related care
	Infection of postoperative incision	T81.415	Yes	No	Persistent wound and wound-related care
	Postoperative skin necrosis	T81.813	Yes	No	Persistent wound and wound-related care
	Postoperative wound dehiscence	T81.31	Yes	No	Persistent wound and wound-related care
	Pyoderma gangrenosum	L88.40	Yes	No	Persistent wound and wound-related care
	Skin ulcer	L98.49xx22	Yes	No	Persistent wound and wound-related care
	Multiple injuries of the ankle	S99.70xx02	Yes	No	Persistent wound and wound-related care
Other	Burns	T31.00xx01	Yes	No	Persistent wound and wound-related care
	Second-degree scald	T30.20xx01	Yes	No	Persistent wound and wound-related care
	Frostbite of the left foot with tissue necrosis	T34.02xx02	Yes	No	Persistent wound and wound-related care
	Frostbite of both feet with tissue necrosis	T34.55xx04	Yes	No	Persistent wound and wound-related care

**Table A2:** ICD-10 definitions, updated disease weights, hierarchy rules, and modifications used to construct the modified Charlson Comorbidity Index (mCCI).

Comorbidity Category	ICD-10 Code(s) (Quan et al., 2005 [27])	Updated Weight (Quan et al., 2011 [28])	Hierarchy Rule	Contribution to mCCI
Myocardial infarction	I21.x, I22.x, I25.2	0	—	0
Congestive heart failure	I09.9, I11.0, I13.0, I13.2, I25.5, I42.0, I42.5–I42.9, I43.x, I50.x, P29.0	2	—	Excluded because of overlap with cardiovascular disease
Peripheral vascular disease	I70.x, I71.x, I73.1, I73.8, I73.9, I77.1, I79.0, I79.2, K55.1, K55.8, K55.9, Z95.8, Z95.9	0	—	Excluded because PAD/PVD was entered separately
Cerebrovascular disease	G45.x, G46.x, H34.0, I60.x–I69.x	0	—	0
Dementia	F00.x–F03.x, F05.1, G30.x, G31.1	2	—	2
Chronic pulmonary disease	I27.8, I27.9, J40.x–J47.x, J60.x–J67.x, J68.4, J70.1, J70.3	1	—	1
Rheumatologic disease	M05.x, M06.x, M31.5, M32.x–M34.x, M35.1, M35.3, M36.0	1	—	1
Peptic ulcer disease	K25.x–K28.x	0	—	Identified but contributes 0
Mild liver disease	B18.x, K70.0–K70.3, K70.9, K71.3–K71.5, K71.7, K73.x, K74.x, K76.0, K76.2–K76.4, K76.8, K76.9, Z94.4	2	Superseded by moderate/severe liver disease	2
Diabetes without chronic complications	E10.0, E10.1, E10.6, E10.8, E10.9; E11.0, E11.1, E11.6, E11.8, E11.9; E12.0, E12.1, E12.6, E12.8, E12.9; E13.0, E13.1, E13.6, E13.8, E13.9; E14.0, E14.1, E14.6, E14.8, E14.9	0	Superseded by complicated diabetes	Excluded
Diabetes with chronic complications	E10.2–E10.5, E10.7; E11.2–E11.5, E11.7; E12.2–E12.5, E12.7; E13.2–E13.5, E13.7; E14.2–E14.5, E14.7	1	Takes precedence	Excluded because diabetes was entered separately
Hemiplegia or paraplegia	G04.1, G11.4, G80.1, G80.2, G81.x, G82.x, G83.0–G83.4, G83.9	2	—	2
Renal disease	I12.0, I13.1, N03.2–N03.7, N05.2–N05.7, N18.x, N19.x, N25.0, Z49.0–Z49.2, Z94.0, Z99.2	1	—	Excluded because renal insufficiency was entered separately
Any malignancy, including lymphoma and leukemia, except malignant neoplasm of skin	C00.x–C26.x, C30.x–C34.x, C37.x–C41.x, C43.x, C45.x–C58.x, C60.x–C76.x, C81.x–C85.x, C88.x, C90.x–C97.x	2	Superseded by metastatic solid tumor	2
Moderate or severe liver disease	I85.0, I85.9, I86.4, I98.2, K70.4, K71.1, K72.1, K72.9, K76.5, K76.6, K76.7	4	Supersedes mild liver disease	4
Metastatic solid tumor	C77.x–C80.x	6	Supersedes non-metastatic malignancy	6
HIV/AIDS	B20.x–B22.x, B24.x	4	—	4

Note: ICD-10 comorbidity definitions were based on the coding algorithms of Quan et al. (2005) [27], and updated disease weights were based on Quan et al. (2011) [28]. For calculation of the mCCI, age points and Charlson components overlapping with diabetes mellitus, peripheral arterial disease, renal insufficiency, and cardiovascular disease were excluded because these variables were entered separately in the multivariable models. Original hierarchy rules were retained where applicable.

**Table A3:** Sensitivity analysis using a reduced condition-specific model excluding length of stay and surgical procedures (analytic N = 713).

Variable	β	95% CI	*p*-Value	Tolerance	VIF
Age, years	0.010	0.006 to 0.014	<0.001	0.865	1.157
Sex	0.040	−0.086 to 0.166	0.531	0.979	1.022
Modified Charlson Comorbidity Index (mCCI)	0.032	−0.025 to 0.089	0.266	0.954	1.049
Diabetes	0.062	−0.096 to 0.220	0.440	0.608	1.644
Peripheral arterial disease	0.131	−0.021 to 0.284	0.091	0.595	1.681
Clinical infection	0.324	0.199 to 0.449	<0.001	0.932	1.073
Renal insufficiency	−0.167	−0.321 to −0.013	0.033	0.734	1.363
Cardiovascular disease	0.254	0.078 to 0.431	0.005	0.867	1.153
Unfavorable/no clear improvement	0.205	0.020 to 0.389	0.030	0.950	1.053
30-day chronic-wound-related readmission	0.112	−0.242 to 0.467	0.534	0.962	1.039
Wound pathogen results (reference: no clear pathogen evidence)					
Single-bacterial result	0.255	0.118 to 0.392	<0.001	0.877	1.141
Complex pathogen result	0.680	0.466 to 0.895	<0.001	0.921	1.086

Note: This reduced model included age, sex, modified Charlson Comorbidity Index (mCCI), diabetes mellitus, peripheral arterial disease, clinical infection, renal insufficiency, cardiovascular disease, clinical outcome, 30-day chronic-wound-related readmission, and wound pathogen-result categories. Length of stay and surgical procedure variables were excluded.
